# Air Distribution in a Fully-Closed Higher Plant Growth Chamber Impacts Crop Performance of Hydroponically-Grown Lettuce

**DOI:** 10.3389/fpls.2020.00537

**Published:** 2020-05-13

**Authors:** Enrique Peiro, Antonio Pannico, Sebastian George Colleoni, Lorenzo Bucchieri, Youssef Rouphael, Stefania De Pascale, Roberta Paradiso, Francesc Gòdia

**Affiliations:** ^1^MELiSSA Pilot Plant – Claude Chipaux Laboratory, Universitat Autònoma de Barcelona, Barcelona, Spain; ^2^Centre d’Estudis I Recerca Espacials, Institut d’Estudis Espacials de Catalunya, Universitat Autònoma de Barcelona, Barcelona, Spain; ^3^Department of Agricultural Sciences, University of Naples Federico II, Portici, Italy; ^4^EnginSoft S.p.A., Bergamo, Italy

**Keywords:** closed loop life support system, computational fluid dynamics, higher plant characterization, hydroponics, *Lactuca sativa* L., mineral composition

## Abstract

The MELiSSA Pilot Plant (MPP) is testing in terrestrial conditions regenerative life support technologies for human exploration in Space. One of its components is a controlled Higher Plant Chamber (HPC) accommodating hydroponic plant cultures. It consists of a 9 m^3^ single closed growth chamber providing adequate environmental conditions for growing plants, enabling the production of food, water and oxygen for the crew. A critical aspect for a reliable HPC performance is to achieve homogeneous air distribution. The initial experiment carried out in the MPP with lettuce as salad crop, showed uneven plant growth throughout the HPC, which was attributed to inadequate air distribution due to non-homogeneous air velocity profile along the inlet-vents. After a detailed computational fluid dynamics (CFD) analysis, the heating, ventilation, and air conditioning subsystem of the HPC was upgraded and a new experiment was carried out in optimized air flow conditions. Nine-day seedlings of lettuce cultivar “Grand Rapids” were transplanted into the HPC and harvested at the end of the growing cycle, where shoot fresh weight, dry biomass, and shoot mineral composition were analyzed. During the experiment, the environmental control system performed remarkably well based on the biometric measurements as well as the mineral composition leading to a vast homogeneous growth. Overall, the results demonstrated the beneficial effect of an adequate air distribution system in HPCs and the effectiveness of CFD-analysis to design properly the gas distribution. The obtained results are of high relevance for life support systems in space involving plants growth.

## Introduction

Controlled ecological life support systems (CELSSs) or bio-regenerative life support systems (BLSSs) have been defined as systems that guarantee human life for long-term in space environments, being able to provide the necessary food sources (Guo et al., 2017). Their main objective is to provide the crew with food, oxygen and water without the need of continuous resupplying from Earth, therefore recovering resources from the waste generated by the crew (Zabel et al., 2016).

MELiSSA (Micro-Ecological Life-Support System Alternative) project was conceived for the development of a regenerative life-support system for long-term space missions (Mergeay et al., 1988). Micro-Ecological Life-Support System Alternative project has five major compartments colonized by (i) thermophilic anoxygenic bacteria, (ii) photo-heterotrophic bacteria, (iii) nitrifying bacteria, (iv) photosynthetic bacteria and higher plants, and (v) the crew, respectively (Gòdia et al., 2004; Lasseur et al., 2010). The Higher Plants Compartment or Chamber (HPC) is an important component of the MELiSSA loop (Favreau et al., 2005; Waters et al., 2005). The complete MELiSSA loop is demonstrated in the MELiSSA Pilot Plant (MPP), at terrestrial conditions and using rats to mock-up the respiration of the crew. The sizing, design, and construction of the different compartments of this Pilot Plant has been performed for a final demonstration target of providing the oxygen required for one human and 20–40% of the required food.

The use of HPC within BLSSs has a double objective: (i) to provide atmosphere regeneration for the respiration and also (ii) to supply fresh food for the crew, which constitutes not only a key point for nutrition but also encompasses beneficial impact on the crew psychological and overall health status (Koga and Iwasaki, 2013). A detailed review of the agricultural systems developed for space addressing the constraints and needs for the improvement of these systems has been recently published by Wheeler (2017). Among these factors, the advances in hydroponics and in illumination by the use of efficient Light Emitting Diode (LED) are highlighted. Moreover, the use of fully controlled plant chambers is an utmost need for a consistent and reproducible food and oxygen production (Haeuplik-Meusburger et al., 2014).

The most relevant environmental variables (air temperature, humidity, light, air flow, pressure, atmosphere gas composition, etc.) have to be controlled in an HPC to sustain optimal plant growth, this being achieved only if plants are cultivated in isolated chambers (Lasseur et al., 2010).

One of the main driving factors for the design of the HVAC system of a HPC is to obtain maximal gas exchange rates, which is essential for plant photosynthesis and consequently for plant growth. In order to achieve this objective, the air velocity inside the canopy should be above 0.2 m s^–1^ (Kitaya et al., 2004) and the air current speed above the canopy should be more than 1.0 m s^–1^ to obtain maximal gas exchange rates (Kitaya et al., 2003). Air flow is important not only for promoting plant growth, but also for water purification via plant transpiration and maintaining healthy conditions of the crops. Lee et al. (2013) found a clear decrease in tipburn symptoms on lettuce cultivars leaves in a closed plant factory, using pre-screened tipburn-sensitive cultivars, when horizontal air velocity was set to 0.28 m s^–1^ or higher, validating the positive effect of air turbulence to prevent tipburn. On the other hand, in the same work, a higher air velocity (1.04 m s^–1^) caused a decrease in plant growth.

Accordingly, the internal air recirculation in the HPC of the MPP was set to an air exchange rate that according to the design would provide an air velocity inside the canopy of about 0.3 m s^–1^.

In order to obtain an air distribution field inside the plant growth region, the internal air circulation was simulated using computational fluid dynamics (CFD) models. Over the past few years, CFD has proven to be a useful tool to estimate air velocity in a reliable and accurate way, improving airflow mal-distributions and asymmetric airflow in closed environments (Martins et al., 2014; Di Perta et al., 2016; Lee et al., 2018). In particular, three-dimensional CFD analyses have been successfully used to predict and improve the air profiles surrounding the growing crops (Zhang et al., 2016).

In order to optimize the input/output of the MELiSSA Higher Plant compartment, an appropriate crop selection is necessary. Hitherto, various species such as cereals, fruit, tubers and leafy vegetables have been tested as potential candidates for food production in space (Wolff et al., 2014). The main selection criteria for these species were their adaptability based on environmental constraints; therefore, plant size, light requirements, harvest index (HI), as well as nutritional value are considered fundamental aspects for the crop selection (Chunxiao and Hong, 2008; Kyriacou et al., 2016, 2017; Wheeler, 2017). Salad crops have a low water uptake/transpiration ratio, short growing cycle, very high HI and require little crew commitment for cultivation. Lettuce is a ubiquitous species among the crops suggested for life support systems as candidate salad crops for near-term missions such as tomato, radish, spinach, chard, and carrot (Wheeler, 2002). Furthermore, it is a very good candidate in terms of space/time efficiency, light/energy use efficiency, HI and handling time, as well as marking the highest score among the selected crops to be cultivated in the Future Exploration Greenhouse (FEG) at Neumayer Station III and in the International Standard Payload Rack (ISPR) on the International Space Station (ISS; Dueck et al., 2016).

Starting from the above considerations, the aim of the current fully controlled experiments was to assess the effects of an improved airflow distribution in the HPC on growth homogeneity, agronomical performance, and mineral profiling of hydroponically-grown lettuce plants.

## Materials and Methods

### Higher Plant Chamber Description

**FIGURE 1:**
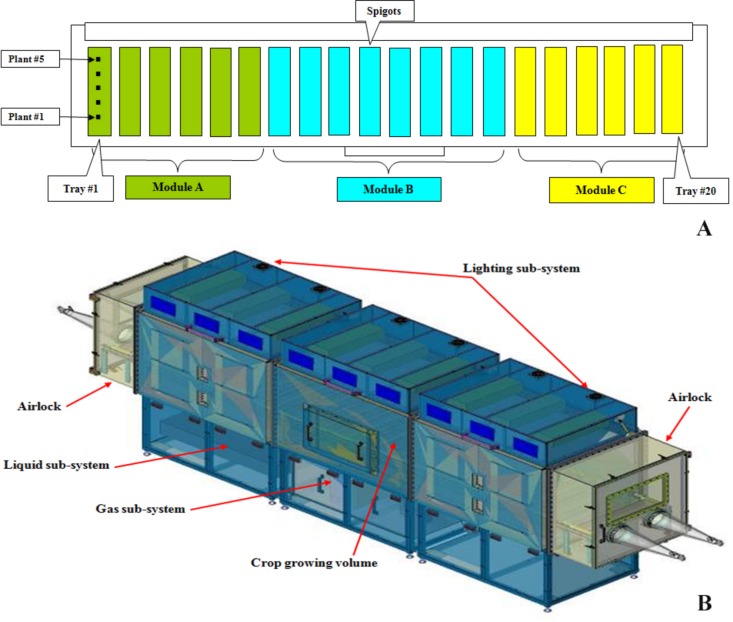
Schematic top view of the growth chamber with plants location and trays numbering **(A)** and hardware representation of the Higher Plant Compartment of MELiSSA Pilot Plant Facilities **(B)**.

The HPC of the MPP was manufactured by the Controlled Environment Systems Research Facility (CESRF – University of Guelph, Ontario, Canada) in collaboration with the company Angstrom Engineering Inc. (Ontario, Canada). It is a closed chamber of the following dimensions: 5.0 m × 1.0 m × 1.2 m (L × W × H), providing a total volume of 6.9 m^3^ for the crops’ growth and a growing surface of around 5 m^2^. It is composed of three modules (A, B, and C) assembled longitudinally (Figure 1). The growing area can host 20 trays. Each tray consisted of five plants (*n* = 100 lettuce plants). For the introduction of new trays and the harvest of already grown plants, two airlocks of 0.69 m^3^ each were installed at both ends (A and C) of the chamber (Figure 1).

**FIGURE 2:**
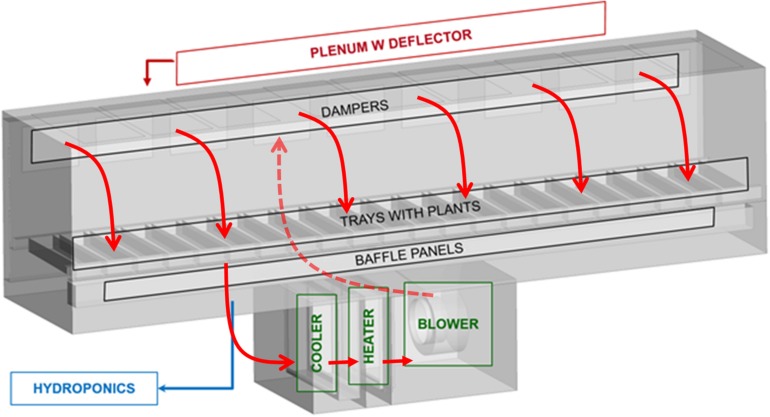
Perspective view of the Higher Plant Compartment, highlighting the air circulation system and components and showing the airflow fluxes (red arrows).

Air recirculation was provided by a centrifugal blower (Centrifugal Exhauster Delhi 410, Canarm, Brockville, Ontario, Canada), controlled by a VFD motor with a controller of 0–50 Hz (SMVector, Lenze, Barcelona, Spain). The airflow was measured in the HVAC plenum by means of an air velocity sensor (8455 TSI, Shoreview, MN, United States). From the plenum, the air was distributed in the chamber through nine louvers with adjustable slats, and was recovered again at the bottom through perforated baffle plates under the trays (Figure 2). Three PTFE bags of 100 L volume each (Keika Ventures, LLC, Chapel Hill, NC, United States), located behind each module, allowed atmospheric pressure compensation. Relative humidity and temperature were controlled in the chamber based on the measurement provided by the humidity and temperature sensors and the action of the dedicated heat exchangers (Delhi CW15, Canarm, Brockville, Ontario, Canada). The first one was controlled by cooling water in order to reduce the excess of humidity, and the second one was supplied by hot water to reach the proper temperature in the chamber. An additional RH/T sensor was located in the air distribution plenum, and additional thermistors (2–3 per module) were provided along the chamber. The generated condensates were collected in a stainless steel 4 L condensate collecting tank and then transferred to the hydroponics nutrient tank. The lighting was provided by six lamps 600 W High pressure sodium (HPS) (HSE 600, P.L. Light Systems, Beamsville, Ontario, Canada), two per each module, and three 400 W Metal Halide (LMH) lamps (MH-400, P.L. Light Systems, Beamsville, Ontario, Canada), one per module, located at the roof of the growing area that presents one glass window per module. For each module, two dedicated fans (MC2483, Comair Rotron, Richardson, TX, United States) were installed to remove the generated heat (keeping the temperature in the lamps loft below 40°C). The gas composition inside the chamber was analyzed by an O_2_/CO_2_ gas analyzer (600 Series, California Analytical, Orange, United States), and CO_2_ concentration was controlled by the injection of CO_2_ until reaching the desired level. The flow was measured by a mass flow controller (EW-32907-67, Cole-Parmer, Vernon Hills, IL, United States), allowing the calculation of the cumulated CO_2_ volume injected.

The hydroponic loop included a 200 L main nutrient reservoir (Central Plastics, Irvine, CA, United States), an irrigation pump with a flow of 0–200 L min^–1^ (Penta KB Drive, Emerson, St. Louis, MO, United States) and a distribution piping composed of four branches that supply the nutrient flow to all the trays through 20 spigots. The hydroponic total flow was measured by a 0–37 L min^–1^ flow sensor (RFO-2500, Gems, Plainville, CT, United States). In a bypass of the main pipe, pH and electrical conductivity (EC) sensors were fixed. The nutrient solution pH and EC were measured by a pH Sensor (HI 8614, Hanna Instruments, Villafranca, Padovana, Italy) and an EC sensor (HI 7638, Hanna Instruments, Villafranca, Padovana, Italy), respectively. The pH control was performed by the PLC according the desired set point through the addition of acid (0.5 M HNO_3_) and base (0.5 M KOH) that were stored in dedicated 10-L tanks. Two concentrated solutions (Stock A and B) were stored also in dedicated 10-L tanks and were injected into the loop in order to control the EC according to the EC set point. The nutrient tank and trays were manufactured from polypropylene, as well as the acid, base, nutrient A and nutrient B tanks (Nalgene, Rochester, NY), while the main collector and the pipelines were manufactured from stainless steel (316-L).

### Computational Fluid Dynamics Models

The numerical models used to simulate the air distribution inside the plant growth chamber were generated using commercial CFD software (CFX, ANSYS Inc., Canonsburg, PA, United States). All the analyses were performed in steady-state conditions. Since the airflow encounters different local flow regimes, the Gamma-Theta transitional laminar-turbulent model was implemented. In this way and based on the local Reynolds number, the flow can switch from fully turbulent (e.g., in the blower region) to laminar (e.g., close to the growing crops). Due to the low temperature variations, air was considered incompressible. As boundary condition, the models imposed a fix value of recirculating mass flow rate, equal to the measured experimental value with nominal blower speed (0.77 kg s^–1^).

Firstly, the CFD study had the objective of replicating the airflow conditions with suboptimal air distribution. Once the air velocity field was obtained in the overall fluid domain, an optimization study was performed to balance the air pattern at growing plants height. In order to identify the best solution, the objective was to minimize the standard deviation of the air velocity in the region of interest.

All 3D CFD models were carried out using identical boundary conditions, in order to compare the internal air distribution at the same operating point.

### Plant Material and Growth Chamber Conditions

About 200 lettuce seeds (*Lactuca sativa* L. cultivar ‘Grand Rapids’, Stokes Seeds Ltd.) were first sanitized with 5% sodium hypochlorite solution for 15 min and after rinsing with demineralized water they were dispersed onto previously autoclaved dark absorbent paper, moist with demineralized water and incubated at room temperature under indirect lighting. After 48 h, seeds having about 1 cm radicle were transferred onto small rockwool cubes (Grodan AO 36/40 6/15W), previously autoclaved and soaked with the nutrient solution, and then they were incubated at 24°C in a dedicated nursery according to a light/dark regime of 16/8 h and a light intensity of 200 ± 50 μmol m^–2^ s^–1^. After seven days, selected seedlings (homogenous second true leaf stage) were transplanted into large sterilized rockwool cubes (Grodan Delta 4G 42/40) presoaked with the nutrient solution, and then placed on the trays of HPC. Five seedlings were placed on each tray, with a total of 20 trays placed into the HPC (100 plants in total). Plants in the HPC were grown according to a light/dark regime of 16/8 h with an intensity of 460 ± 60 μmol m^–2^ s^–1^, while temperature and relative humidity were regulated at 26/20°C and 50/70%, respectively. The experiment was carried out with a CO_2_ concentration set at 1000 ppm.

Lettuce plants were cultivated in a Nutrient Film Technique (NFT) growing system (closed loop hydroponic system). The trays were 80 cm long, 15 cm wide and 8 cm deep, having an inclination of 1%. Each tray was covered with perforated stainless steel covers to avoid the algae proliferation and to limit the nutrient solution evaporation. The flow rate of the nutrient solution was set at 1.5 L min^–1^, supplied at the top end of each tray, while the excess was gathered in the general collector and subsequently in the reservoir tank.

The composition of the nutrient solution was as follows: 3.62 mM Ca(NO_3_)_2_ ⋅ 4H_2_O, 0.08 mM FeCl_3_ ⋅ 6H_2_0, 0.1 mM Na ⋅ EDTA, 1 mM MgSO_4_ ⋅ 7H_2_O, 5 mM KNO_3_, 1.5 mM NH_4_H_2_PO_4_, 1 mM (NH_4_)_2_SO_4_, 0.02 mM H_3_BO_3_, 5 μM MnSO_4_ ⋅ H_2_O, 3.5 μM ZnSO_4_ ⋅ 7H_2_O, 0.8 μM CuSO_4_ ⋅ 5H_2_O, and 0.5 μM H_2_MoO_4_ (85% MoO_3_). The nutrient solution was prepared starting from 100 times concentrated Stock A (Ca(NO_3_)_2_ ⋅ 4H_2_O, FeCl_3_ ⋅ 6H_2_0 and Na ⋅ EDTA) and Stock B (rest of the components) solutions, while the nutrient solution pH and EC were automatically managed by the control system maintaining a set point of 5.9 ± 0.1 and 1.9 ± 0.05 dS m^–1^, respectively. The nutrient solution was completely replaced every 2 weeks. The harvesting of all the plants was performed at 19 and 28 days after transplanting (DAT) in the HPC for Test 1 and Test 2, respectively.

### Biomass Determination and Leaf Mineral Analysis

At the end of each test, all plants were harvested and separated into edible and inedible fractions on a plant basis. Shoot fresh weight per plant was determined immediately after chamber opening. Shoot and roots of each plant were oven dried at 70°C for three days, until reaching a constant weight and then weighed for dry biomass determination.

The relative growth rate (RGR) was calculated based on the following formula described by Hunt et al. (2002):

$$\text{RGR}=\left(\log_{e}W_{2}-\log_{e}W_{1}\right)/\left(t_{2}-t_{1}\right)$$

where *t* is time (*t*_1_ transplant day; *t*_2_ harvest day), *W*_1_ shoot dry weight at transplant and *W*_2_ shoot dry weight at harvest.

Determinations of morphological traits: plant and roots dry weight, fresh and dry shoot weight were conducted at harvest on all five plants of the experimental unit (i.e., tray) and were averaged to produce the replicate mean (*n* = 20). The dry biomass produced from these lettuce plants was further used as an aggregate replicate sample for analysis of mineral composition (*n* = 20).

Dried leaf samples were ground separately with an electrical mill to an 841 μm screen, then 0.25 g of the dried tissues were analyzed for mineral content by ion chromatography: P, K, Ca, and Mg as described in detail by Rouphael et al. (2017) and Kyriacou et al. (2019). Nitrogen (total N) concentration in the shoot tissues was determined on 1 g of dried samples by Kjeldahl method (Bremner, 1965).

## Results and Discussion

### Test Rationale

The two tests conducted in our study reflect the need to have a proper distribution of the gas phase in a plant chamber in order to guarantee uniform plant growth throughout the overall cultivation area of the HPC. Both tests shared the same methodology in terms of plant material, growth conditions and biomass analysis as described above, as well as the plant harvest scheduled at 28 DAT. However, during Test 1, following the daily routine visual inspections, an evident heterogeneity of plant size was noted starting from the beginning of the third week after transplanting. This lack of homogeneity showed a different plant growth stage between the HPC modules, in particular all plants in the growth chamber were at the phenological phase of head development (Stage 4) according to *Biologische Bundesanstalt, Bundessortenamt and CHemical industry* (BBCH) scale for leaf vegetables (Meier, 2001), but about 70% of lettuce plants had already reached the full maturity (Code 49 of BBCH-scale). Therefore, in order to avoid the early achievement of the phenological flowering phase (Code 50 of BBCH-scale), the experiment was terminated earlier than expected and the lettuce plants were harvested at 19 DAT to further analyze potential causes of the visually evident non-homogeneous growth. Thus, it was identified that there was a lack of homogeneous distribution of the gas phase in the chamber as well as not appropriate circulation regime. Consequently, CFD tools were used to guide the design of the hardware modifications in order to solve this malfunction. After the corresponding changes in the HPC, Test 2 was conducted in which a higher homogeneity of growth compared to Test 1 was detected and all the plants in the chamber reached the full maturity (Code 49 of BBCH-scale) at 28 DAT as scheduled in our test plan protocol.

### CFD Analysis and Technical Modifications Performed in the Airflow System of the Plant Chamber

**FIGURE 3:**
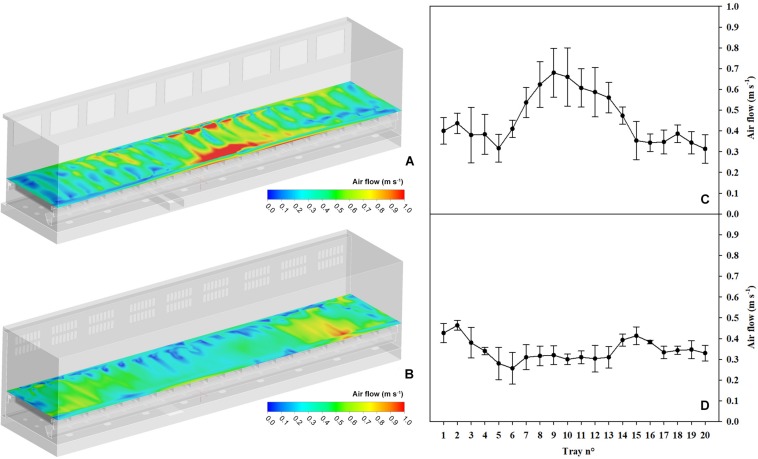
Computational Fluid Dynamics (CFD) analysis of the airspeed at the canopy level along the Higher Plant Chamber (HPC) surface and average airspeed at the canopy level per tray in Test 1 (**A,C**, respectively) and Test 2 (**B,D**, respectively).

CFD analyses enabled the determination of the complete fluid dynamics field inside the plant growth chamber, studying the suboptimal air distribution of the original configuration of the chamber. The results assessment was primarily based on the investigation of the air velocity magnitude at plant height, in order to have complete information regarding the environment in which the crop was grown. With suboptimal air distribution (Test 1), the air velocity was significantly higher in the trays located in the central part of the chamber, where the magnitude approached 1.0 m s^–1^ (Figure 3A). The air velocity was averaged for the five plants per tray (Figure 3C). Particularly, the central trays (tray no. 7 to tray no. 14) had almost the double velocity value of that at the side trays (about 0.35 and 0.6–0.7 m s^–1^ in the side and central trays, respectively).

In order to improve the chamber homogeneity for the gas phase, the CFD analysis guided changes in several elements of the heating, ventilation and air conditioning subsystem of the chamber, as follows:

a) The plenum region was modified by the insertion of a deflector, a stainless steel perforated baffle designed in order to deviate the air flow from the central region and obtain a more uniform air distribution at the plenum exit.
b) In order to achieve control on the supply air flow to the growing region, new dampers were installed connecting the plenum region to the chamber. Such components are adjustable, able to modify their open area to allow more or less crossing air flow. Nine stainless steel dampers were manufactured with dimensions 350 mm × 450 mm, guaranteeing the flexibility of the air distribution system.
c) In the return air system, the baffle panels’ configuration was modified. The arrangement of the baffle panels was based on the original configuration, considered not adequate for a sufficient return air uniformity. The baffle panels’ configuration was redesigned, with different hole patterns on the baffles.
d) Finally, in the original configuration the baffle panels were not positioned against the side walls, leaving open gaps where air could bypass the panels. In order to force the air flow through the baffle panels, 10 additional stainless steel components were designed and manufactured closing the side gaps.

With these changes, CFD models were predicting an improved balance throughout the 20 trays with an average air velocity of 0.34 m s^–1^ (Figures 3B,D), corroborating that in the improved configuration, the air velocity increased its evenness at plant height, with the same overall flow rate (averaged for the five plants per tray, Figure 3D). Once the changes in the chamber had been implemented, a new test (Test 2) was performed.

### Assessment of Plant Biomass Distribution Along the HPC and Shoot Mineral Composition in the Two Plant Cultivation Tests

**FIGURE 4:**
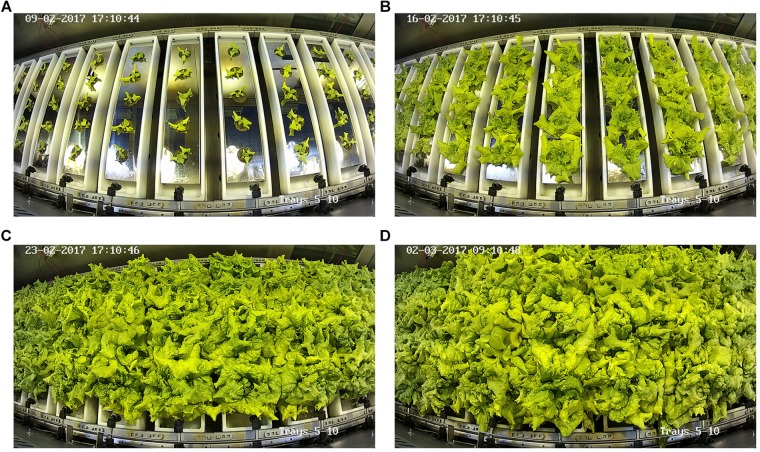
Evolution of lettuce plants growth over the weeks (**A, B, C** and **D**, respectively for 7, 14, 21, and 28 DAT) during Test 2, captured by one of the internal cameras of the HPC.

The growth parameters measured at harvest in both Tests 1 and 2 are reported in Table 1. Considering the different duration of the two tests, in Test 1 the lettuce plants were harvested 19 DAT, while in Test 2 the harvest occurs 28 DAT (Figure 4); plant roots and shoot fresh and dry weight at harvest were significantly higher in the second test compared to the first one (Table 1). On the other hand, the RGR was not statistically different in the two tests, this result can be explained considering that the plants in both tests were harvested during the same phenological phase of head development (Stage 4 of BBCH-scale) and therefore long before the stationary growth phase was reached which coincides with the beginning of the flowering phase (Code 50 of BBCH-scale; Meier, 2001). However, the HI was significantly higher in Test 2 than in Test 1 (Table 1), while the plant biomass in Test 1 was about 25% lower than the reference values obtained from several tests performed under comparable environmental conditions and crop growth duration (Hanford, 2006; Wheeler and Sager, 2006). The harvest in Test 2 occurred 9 days later compared to Test 1, and the fresh and dry weight per plant were obviously higher in the second test, but in any case the shoot dry biomass in Test 2 was about 25% higher than that obtained by Wheeler et al. (1994) after 28 DAT on a different cultivar, but in completely comparable environmental conditions.

**TABLE 1:** Plant dry weight, roots dry weight, shoot dry and fresh weight, harvest index, relative growth rate (RGR), shoot dry weight standard deviation (SD) and shoot dry weight standard deviation percentage of hydroponically-grown lettuce plants in Test 1 and Test 2.

Test	Plant dry weight (g plant^–1^)	Roots dry weight (g plant^–1^)	Shoot dry weight (g plant^–1^)	Shoot fresh weight (g plant^–1^)	Harvest index	RGR (mg mg^–1^ day^–1^)	Shoot dry weight (SD)	Shoot dry weight (SD%)
Test 1	5.13 ± 0.26	0.73 ± 0.04	4.40 ± 0.23	87.42 ± 4.61	0.86 ± 0.001	0.089 ± 0.003	1.01	23.01
Test 2	12.68 ± 0.35	1.05 ± 0.05	11.63 ± 0.33	278.61 ± 6.19	0.92 ± 0.003	0.083 ± 0.001	1.46	12.59
Student’s *t*-test	***	***	***	***	***	ns	–	–

All data are expressed as mean ± standard error, n = 20. ns, *** Non-significant or significant at *P* ≤ 0.001, respectively. SD: standard deviation.

**FIGURE 5:**
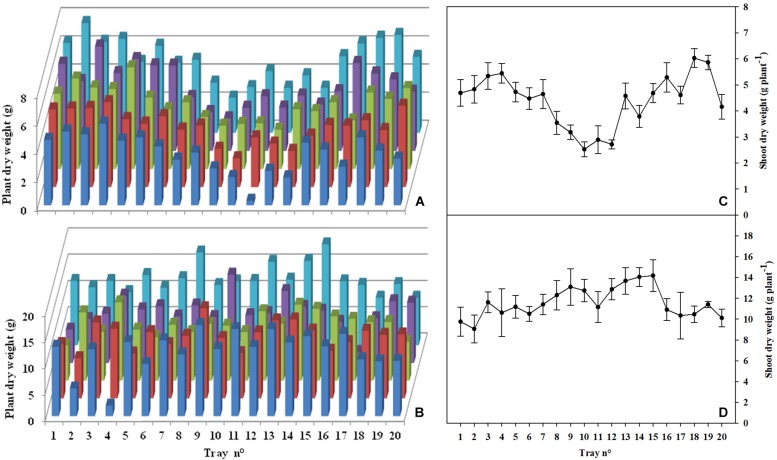
3D plant dry biomass distribution (*n* = 100) and shoot dry weight distribution (*n* = 20) along the Higher Plant Chamber (HPC) in Test 1 (**A,C**, respectively) and Test 2 (**B,D**, respectively).

**FIGURE 6:**
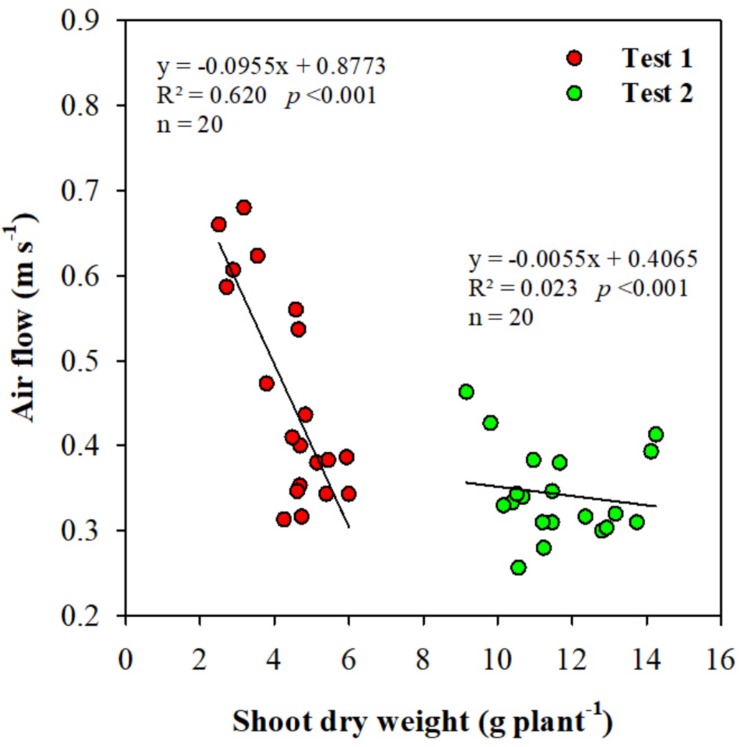
Regression between the air flow velocity at the different trays position (*n* = 20) of the Higher Plant Chamber (HPC) and the respective shoot dry weight (*n* = 20) in Test 1 and Test 2.

Regarding the distribution of the shoot dry weight along the HPC, the value of the standard deviation has considerably decreased in Test 2 with respect to Test 1 (12.59 and 23.01, respectively; Table 1). Plant dry biomass and shoot dry weight distribution along the growth chamber in both tests are presented in Figure 5. Comparing the distribution of plant growth along the chamber in the two tests, a clear lack of uniformity was noted in Test 1, both in terms of shoot and total dry biomass (Figures 5A,C). An evident decrease in shoot dry weight was observed starting the tray no. 8 until the tray no. 14. In particular, a 33.5% average reduction of the shoot dry weight in the seven central trays was recorded with respect to the plants positioned on the lateral trays of the HPC (Figure 5C). Test 2 clearly shows a better uniformity of plant dry biomass distribution and a greater homogeneity of the average shoot dry weight per tray along the growth chamber (Figures 5B,D). In addition, the lettuce plants of Test 2 recorded a HI of 0.92, in agreement with data reported by Wheeler and Sager (2006) in several experiments of lettuce grown in a fully-closed growth chamber in comparable conditions. Instead, the significantly lower HI obtained in the first experiment (0.86), denotes a stress condition at the canopy level leading to an imbalance of the shoot to roots ratio. An appropriate air circulation inside a growth chamber promotes growth and maximizes gas exchange rates (Kitaya et al., 1998). In Test 1, an air velocity higher than 0.5 m s^–1^ at the level of the central trays of the HPC (Figure 3C) has resulted in a reduction of the shoot dry weight in correspondence of the same positions of the chamber (Figure 5C). This result was statistically confirmed by the significant inverse linear regression (*R*^2^ = 0.62) existing between the air flow at the different trays position of the chamber and the respective shoot dry weight of Test 1 plants (Figure 6). On the contrary, in Test 2 the poor correlation, confirmed by the low value of the coefficient of determination (*R*^2^ = 0.02), indicated that the shoot dry weight was not affected by the air velocity inside the chamber (Figure 6). These findings comply with what was found in the work of Lee et al. (2013), in which the lettuce cultivar “Dambaesangchuesse” observed a significant decrease in leaf area and shoot fresh weight at an air velocity greater than 0.55 m s^–1^. Likewise, in another work carried out in a plant factory on “Greenwave” lettuce cultivar, a reduction in the dry weight of the shoots was observed at an air velocity greater than 0.9 m s^–1^ (Nishikawa et al., 2013). Kitaya et al. (2003) found in a work with sweet potato, that the net photosynthetic rate and the transpiration rate increased significantly as the air velocity increased from 0.01 to 0.2 m s^–1^, but further increases of air speed up to 1.0 m s^–1^ gradually increased transpiration rate, while the photosynthetic rate remained rather constant. Moreover, in a similar work with tomato, a decrease in the photosynthetic rate was found following an increase in air velocity from 0.4 to 0.8 m s^–1^ (Kitaya et al., 2004). In lettuce, on the other hand, it was observed that an air velocity of 0.7 m s^–1^ caused water stress followed by the reduction of stomatal conductance (Shibata et al., 1995). Based on these findings and the results from Test 2, we can deduce that in a closed growth chamber, a homogeneous air velocity between 0.3 and 0.5 m s^–1^ enables to optimize gas exchange rates and consequently promotes lettuce plants growth and more importantly plants growth uniformity in the chamber.

Shoot mineral composition of both harvests are presented in Table 2. Among the minerals analyzed, K was by far the most abundant, regardless of the two tests, followed by N, P, Ca, and Mg. The nitrogen content was higher in the second test, reaching values of 5.8 g 100 g^–1^ dw compared to 5.2 g 100 g^–1^ dw in Test 1. Contrarily, the concentration of potassium and calcium had slightly decreased in Test 2 with respect to Test 1, where potassium content reached 7.3 and 6.9 g 100 g^–1^ dw and calcium content reached 0.9 and 0.6 g 100 g^–1^ dw, in Test 1 and 2, respectively. However, phosphorus and magnesium content was instead fully comparable in both tests.

**TABLE 2:** Comparison of shoot mineral copmposition of hydroponically-grown lettuce plants coming from Test 1 and Test 2 with scientific literature.

Test	N (g 100 g^–1^ dw)	P (g 100 g^–1^ dw)	K (g 100 g^–1^ dw)	Ca (g 100 g^–1^ dw)	Mg (g 100 g^–1^ dw)
Test 1	5.2	0.9	7.3	0.9	0.2
Test 2	5.8	0.8	6.9	0.6	0.2
Wheeler et al. (1994)	4.8	0.4	17.0	0.9	0.3
McKeehen et al. (1996)	4.5	0.6	8.2	0.6	0.2
Masot Mata (2007)	5.5	1.0	7.9	1.2	0.3
Rouphael et al. (2019)	–	0.5	6.5	0.8	0.3

*Test 1 and Test 2 data are expressed as mean, n = 20. Dw, dry weight basis.*

The reduction of K and Ca content recorded in Test 2 compared to Test 1 could be a result of a higher transpiration rate that occurred in the first test following the higher air velocity measured in the central position of the HPC. In fact, the increase in transpiration rate promotes the transport of macronutrients toward the leaves; in particular Goto and Takakura (1992) had found in several works on lettuce, an increase in calcium foliar content following the increase in air speed at canopy level. Conversely, the increase in shoot nitrogen content in Test 2 compared to Test 1 can be related to the growing stage, as well as to the size of the plant. Indeed, in a test carried out in an open-gas-exchange growth chamber on two butterhead lettuce cultivars, it has been observed that the shoot nitrogen content during the growing period increased with the increase of the plant fresh biomass, according to a highly significant positive quadratic correlation (El-Nakhel et al., 2019). Regardless the different air velocity conditions of the two tests, the shoot mineral content appears to be almost in line with the literature data referring to the lettuce cultivation in closed or semi-closed growth chambers (Table 2). The only notable difference was observed in the work of Wheeler et al. (1994), in which higher potassium content and a lower concentration of phosphorus are reported, probably due to different growing conditions rather than to genetic factors.

## Conclusion

The successful cultivation of higher plants in a growth chamber is strongly related to the precise control of environmental variables. To sustain optimal plant growth, it is essential to maximize gas exchange rates especially in a fully closed growth chamber. One of the main factors that strongly affects the gas exchanges and consequently the plant growth is the air velocity at the canopy level. Furthermore, the movement of air is important not only to enhance plant growth, but also for effective water recycling through plant transpiration in human life support systems. Especially in limiting conditions, such as space outposts, it is important to ensure a homogeneous distribution of biomass along the growth chamber, in order to achieve efficient use of resources. The results of this study indicate that air velocities between 0.3 and 0.5 m s^–1^ maximizes biomass production in the HPC, while air speed values above 0.6 m s^–1^ can depress the lettuce plant growth. Finally, a homogeneous air circulation at the canopy level guarantees uniform distribution of shoot biomass along the growth chamber.

## Data Availability Statement

The datasets generated for this study are available on request to the corresponding author.

## Author Contributions

EP, AP, and FG coordinated the whole project, provided the intellectual input and set up the experiment. EP and FG conducted the Test 1 and AP the Test 2 and wrote the Introduction and a section of the Materials and Methods. SC and LB wrote the parts relative to CFD models. AP and YR were involved in writing agronomical data analysis and data interpretation. AP wrote the parts relative to the lettuce crop performance. EP, AP, YR, and FG critically revised the final draft of the manuscript. All authors approved the final draft preparation.

## Conflict of Interest

The authors declare that the research was conducted in the absence of any commercial or financial relationships that could be construed as a potential conflict of interest.

## References

[B2] Bremner J. M. (1965). “Total nitrogen,” in *Methods of Soil Analysis*, eds Black C. A. Evans D. D. White I. L. Ensminger L. E. Clark F. E. (Madison, WI: American Society of Agronomy), 1149–1178. 10.2134/agronmonogr9.2.2ed.c32

[B3] Chunxiao X. Hong L. (2008). Crop candidates for the bioregenerative life support systems in China. *Acta Astronaut.* 63 1076–1080. 10.1016/j.actaastro.2008.02.003

[B4] Di Perta E. S. Agizza M. A. Sorrentino G. Boccia L. Pindozzi S. (2016). Study of aerodynamic performances of different wind tunnel configurations and air inlet velocities, using computational fluid dynamics (CFD). *Comp. Elect. Agric.* 125 137–148. 10.1016/j.compag.2016.05.007

[B5] Dueck T. Kempkes F. Esther M. Cecilia S. (2016). “Choosing crops for cultivation in space,” in *Proceedings of the 46th International Conference on Environmental Systems*, Vienna.

[B6] El-Nakhel C. Giordano M. Pannico A. Carillo P. Fusco G. M. De Pascale S. (2019). Cultivar-specific performance and qualitative descriptors for butterhead *Salanova lettuce* produced in closed soilless cultivation as a candidate salad crop for human life support in space. *Life* 9:61. 10.3390/life9030061 31337144 PMC6789809

[B7] Favreau M. Ordonez L. Rodriguez A. Waters G. (2005). “Application of non-rectangular hyperbola model to the lettuce and beet crops,” in *Proceedings of the G. 05ICES-23, 35th ICES*, Rome.

[B8] Gòdia F. Albiol J. Perez J. Creus N. Cabello F. Montras A. (2004). The MELISSA pilot plant facility as an integration test-bed for advanced life support systems. *Adv. Space Res.* 34 1483–1493. 10.1016/j.asr.2003.08.038 15846877

[B9] Goto E. Takakura T. (1992). Prevention of lettuce tipburn by supplying air to inner leaves. *Trans. ASAE* 35 641–645. 10.13031/2013.28644

[B10] Guo S. S. Mao R. X. Zhang L. L. Tang Y. K. Li Y. H. (2017). Progress and prospect of research on controlled ecological life support technique. *Reach* 6 1–10. 10.1016/j.reach.2017.06.002

[B11] Haeuplik-Meusburger S. Paterson C. Schubert D. Zabel P. (2014). Greenhouses and their humanizing synergies. *Acta Astronaut.* 96 138–150. 10.1016/j.actaastro.2013.11.031

[B12] Hanford A. J. (2006). *Advanced Life Support Baseline Values And Assumptions Document. Technical Reports, 3.* Available online at: http://docs.lib.purdue.edu/nasatr/3 (accessed October 10, 2019).

[B13] Hunt R. Causton D. R. Shipley B. Askew A. P. (2002). A modern tool for classical plant growth analysis. *Ann. Bot.* 90 485–488. 10.1093/aob/mcf214 12324272 PMC4240380

[B14] Kitaya Y. Shibuya T. Kozai T. Kubota C. (1998). Effects of light intensity and air velocity on air temperature, water vapor pressure, and CO2 concentration inside a plant canopy under an artificial lighting condition. *Life Supp. Bios. Sci.* 5 199–203. 11541677

[B15] Kitaya Y. Shibuya T. Yoshida M. Kiyota M. (2004). Effects of air velocity on photosynthesis of plant canopies under elevated CO2 levels in a plant culture system. *Adv. Space Res.* 34 1466–1469. 10.1016/j.asr.2003.08.031 15825257

[B16] Kitaya Y. Tsuruyama J. Shibuya T. Yoshida M. Kiyota M. (2003). Effects of air current speed on gas exchange in plant leaves and plant canopies. *Adv. Space Res.* 31 177–182. 10.1016/s0273-1177(02)00747-0 12578005

[B17] Koga K. Iwasaki Y. (2013). Psychological and physiological effect in humans of touching plant foliage - using the semantic differential method and cerebral activity as indicators. *J. Physiol. Anthropol.* 32 1–9. 10.1186/1880-6805-32-7 23587233 PMC3660240

[B18] Kyriacou M. C. De Pascale S. Kyratzis A. Rouphael Y. (2017). Microgreens as a component of space life support systems: a cornucopia of functional food. *Front. Plant Sci.* 8:1587. 10.3389/fpls.2017.01587 28955372 PMC5600955

[B19] Kyriacou M. C. El-Nakhel C. Graziani G. Pannico A. Soteriou G. A. Giordano M. (2019). Functional quality in novel food sources: genotypic variation in the nutritive and phytochemical composition of thirteen microgreens species. *Food Chem.* 277 107–118. 10.1016/j.foodchem.2018.10.098 30502125

[B20] Kyriacou M. C. Rouphael Y. Di Gioia F. Kyratzis A. Serio F. Renna M. (2016). Micro-scale vegetable production and the rise of microgreens. *Trends Food Sci. Technol.* 57 103–115. 10.1016/j.tifs.2016.09.005

[B21] Lasseur C. Brunet J. de Weever H. Dixon M. Dussap G. Godia F. (2010). MELiSSA: the European Project of closed life support system. *Gravitat. Space Biol.* 23 3–12.

[B22] Lee J. G. Choi C. S. Jang Y. A. Jang S. W. Lee S. G. Um Y. C. (2013). Effects of air temperature and air flow rate control on the tipburn occurrence of leaf lettuce in a closed-type plant factory system. *Hortic. Environ. Biotechnol.* 54 303–310. 10.1007/s13580-013-0031-0

[B23] Lee M. S. Li Z. Ling J. Aute V. (2018). A CFD assisted segmented control volume based heat exchanger model for simulation of air-to-refrigerant heat exchanger with air flow mal-distribution. *Appl. Thermal Eng.* 131 230–243. 10.1016/j.applthermaleng.2017.11.094

[B24] Martins N. M. Carrico N. J. Ramos H. M. Covas D. I. (2014). Velocity-distribution in pressurized pipe flow using CFD: accuracy and mesh analysis. *Comp. Flui.* 105 218–230. 10.1016/j.compfluid.2014.09.031

[B25] Masot Mata A. (2007). *Engineering Photosynthetic Systems For Bioregenerative Life Support.* Ph. D. thesis, Universitat Autònoma de Barcelona, Bellaterra.

[B26] McKeehen J. D. Smart D. J. Mackowiak C. L. Wheeler R. M. Nielsen S. S. (1996). Effect of CO2 levels on nutrient content of lettuce and radish. *Ad. Space Res.* 18 85–92. 10.1016/0273-1177(95)00864-b 11538818

[B27] Meier U. (2001). *Growth Stages Of Mono- And Dicotyledonous Plants. Bbch Monograph. German Federal Biological Research Centre For Agriculture And Forestry.* Hoboken, NJ: Blackwell Science.

[B28] Mergeay M. Verstraete W. Dubertret G. Lefort-Tran M. Chipaux C. Binot R. A. (1988). “MELiSSA’—A micro-organisms-based model for ‘CELSS’ development,” in *Proceedings at the 3rd European Symposium on Space Thermal Control Life Support Systems Noordwijk*, Piscataway, NJ.

[B29] Nishikawa T. Fukuda H. Murase H. (2013). Effects of airflow for lettuce growth in the plant factory with an electric turntable. *IFAC Proc.* 46 270–273. 10.3182/20130327-3-jp-3017.00062

[B30] Rouphael Y. Colla G. Giordano M. El-Nakhel C. Kyriacou M. C. De Pascale S. (2017). Foliar applications of a legume-derived protein hydrolysate elicit dose-dependent increases of growth, leaf mineral composition, yield and fruit quality in two greenhouse tomato cultivars. *Sci. Hortic.* 226 353–360. 10.1016/j.scienta.2017.09.007

[B31] Rouphael Y. Petropoulos S. A. El Nakhel C. Pannico A. Kyriacou M. C. Giordano M. (2019). Reducing energy requirements in future bioregenerative life support systems (BLSSs): performance and bioactive composition of diverse lettuce genotypes grown under optimal and suboptimal light conditions. *Front. Plant Sci.* 10:1305. 10.3389/fpls.2019.01305 31736990 PMC6831738

[B32] Shibata T. Iwao K. Takano T. (1995). Effect of vertical air flowing on lettuce growing in a plant factory. *Green Environ. Control Autom.* 399 175–182. 10.17660/actahortic.1995.399.20

[B33] Waters G. Zheng Y. Gidzinski D. Dixon M. (2005). *Empirical Relationships Between Light Intensity And Crop Net Carbon Exchange Rate At The Leaf and FULL CANOPY SCALE: Towards the Integration Of A Higher Plant Chamber In The MELiSSA Loop, - 05ICES-290, 35th ICES.* Guelph: University of Guelph.

[B34] Wheeler R. M. (2002). “Horticulture for mars,” in *Proceedings of the XXVI International Horticultural Congress: Horticulture, Art And Science For Life- Colloquia Presentations*, Toronto, ON.

[B35] Wheeler R. M. (2017). Agriculture for space: people and places paving the way. *Open Agric.* 2 14–32. 10.1515/opag-2017-0002

[B36] Wheeler R. M. Mackowiak C. L. Sager J. C. Yorio N. C. Knott W. M. Berry W. L. (1994). Growth and gas exchange by lettuce stands in a closed, controlled environment. *J. Amer. Soc. Hortic. Sci.* 119 610–615. 10.21273/jashs.119.3.610 11538197

[B37] Wheeler R. M. Sager J. C. (2006). *Crop Production For Advanced Life Support Systems. Technical Reports, 1.* Available online at: http://docs.lib.purdue.edu/nasatr/1 (accessed November 8, 2019).

[B38] Wolff S. Coelho L. Karoliussen I. Jost A. I. (2014). Effects of the extraterrestrial environment on plants: recommendations for future space experiments for the MELiSSA higher plant compartment. *Life* 4 189–204. 10.3390/life4020189 25370192 PMC4187168

[B39] Zabel P. Bamsey M. Schubert D. Tajmar M. (2016). Review and analysis of over 40 years of space plant growth systems. *Life Sci. Space Res.* 10 1–16. 10.1016/j.lssr.2016.06.004 27662782

[B40] Zhang Y. Kacira M. An L. (2016). A CFD study on improving air flow uniformity in indoor plant factory system. *Biosyst. Engin.* 147 193–205. 10.1016/j.biosystemseng.2016.04.012

